# The Utility of Measures of Physical Behavior, Function, and Fitness as Predictors of Mortality

**DOI:** 10.1016/j.mayocpiqo.2026.100710

**Published:** 2026-03-17

**Authors:** Yuhe Wang, Cameron Razieh, Alex V. Rowlands, Kishan Bakrania, Richard Russell, Kamlesh Khunti, Melanie J. Davies, Francesco Zaccardi, Thomas Yates

**Affiliations:** a Diabetes Research Centre, University of Leicester, UK; b Leicester Real World Evidence Unit, Diabetes Research Centre, Leicester General Hospital, University of Leicester, Leicester, UK; c NIHR Leicester Biomedical Research Centre, University Hospitals of Leicester NHS Trust and University of Leicester, UK; d Global Research and Development, RGA UK Services Limited, London, UK; e NIHR Applied Research Collaborations – East Midlands, University Hospitals of Leicester NHS Trust, UK

## Abstract

**Objective:**

To assess whether simple measures of lifestyle and physical health improve mortality risk prediction in healthy adults and those living with health conditions when added to or substituted for traditional risk factors.

**Patients and Methods:**

Data were from the UK Biobank (N=407,569; median age, 58 years; May 1, 2006 to September 30, 2022), stratified by sex and health status. The base model included the following: age, smoking status, body mass index, systolic blood pressure (SBP), total cholesterol-to-high-density lipoprotein cholesterol ratio (CHR), and material deprivation. Five simple physical measures (leisure time physical activity; sleep duration; resting heart rate; maximum handgrip strength; and walking pace [WP]) were added to or substituted for traditional risk factors (ie, SBP and CHR), both individually and combined. Model performance was assessed using the C-index and net reclassification index (NRI).

**Results:**

Replacing both CHR and SBP with all physical measures improved C-index of 0.022 (95% CI, 0.018-0.026) and NRI by 9.6% (95% CI, 6.5%-12.8%) in women living with a health condition. Corresponding values for men were as follows: C-index: 0.024 (95% CI, 0.020-0.027); NRI: 19.0% (95% CI, 16.6%-21.4%). The improvement of C-index was smaller in healthy women (0.006; 95% CI, 0.004-0.007) and men (0.007; 95% CI, 0.006-0.009). WP alone improved risk prediction and classification when replacing CHR and SBP in individuals with a health condition, with a C-index improvement of 0.015 (95% CI, 0.012-0.019) and an NRI of 11.0% (95% CI, 7.4%-14.6%) in women and 0.014 (95% CI, 0.012-0.017) and 14.0% (95% CI, 11.5%-16.5%) in men, respectively.

**Conclusion:**

Measures of lifestyle and physical health improve mortality prediction and classification and could be used as potential substitutes for traditional clinical factors, particularly in populations with prevalent illness. WP was the strongest individual predictor.

Risk prediction models are widely used within clinical practice, public health and the insurance sector. From a clinical perspective, risk scores help stratify patients for better personalized decision making.^1^ In public health, risk scores help identify sections of the population that may benefit from preventive interventions.^2^ Life insurance companies harness prediction tools to classify individuals according to their risk of a mortality event to compensate for a liability or loss of income in response a future event that cannot be predicted with certainty.^3^

Traditional risk prediction models typically include nonmodifiable demographic factors (such as sex, age, and a history of chronic disease) and basic modifiable clinical and lifestyle factors (such as blood pressure, cholesterol, smoking status, and body mass index [BMI]).^3^, ^4^, ^5^, ^6^, ^7^ For example, the Framingham Risk Score includes age, sex, total cholesterol, high-density lipoprotein (HDL) cholesterol, systolic blood pressure (SBP), antihypertensive treatment use, smoking status, and diabetes status as key predictors.^8^ Although originally developed to estimate 10-year cardiovascular disease (CVD) risk, CVD risk scores also predict all-cause mortality.^9^^,^^10^

Beyond established risk factors, there has been mounting interest in the utility of easy-to-measure physical behaviors and markers of physical function and fitness for mortality risk prediction and insurance underwriting.^11^^,^^12^ Resting heart rate (RHR), for example, serves as a proxy for fitness level that is inversely associated with both physical fitness^13^ and all-cause mortality.^14^^,^^15^ Similarly, a meta-analysis summarized the inverse association between sleep duration and all-cause mortality.^16^ Other physical activity or function measures, including physical activity,^12^^,^^17^^,^^18^ handgrip strength,^19^^,^^20^ and walking pace (WP),^21^^,^^22^ have been found to have associations with mortality risk. In particular, WP has emerged as a strong predictor of mortality risk, with some studies suggesting that it outperforms cholesterol and blood pressure in risk prediction of all-cause mortality.^23^^,^^24^ These measures are widely used as established markers of physical health and components of frailty indices for unhealthy populations. For example, low physical activity, weak grip strength, and slow walking speed comprise 3 of the 5 criteria of Fried Frailty Index, which was developed to identify frailty in older adults owing to aging or in people with comorbidities.^25^ Frailty indices have also been adapted with claims data,^26^ applied to predict adverse clinical outcomes,^27^ and recommended for inclusion in clinical risk models.^28^

Despite this, limited research has systematically examined the ability of physical behavior, function, and fitness measures to enhance established risk score models, either individually or in combination,^23^^,^^24^ or serve as alternatives to clinical measures such as SBP or cholesterol. It is also unclear whether the prognostic discrimination of these measures is consistent in healthy individuals and in those living with existing health conditions, which is important in an aging population increasingly living with chronic diseases.^29^^,^^30^

The aim of this study was therefore to quantify whether measures of physical behavior (leisure time physical activity [LTPA] and sleep duration) and markers of physical function and fitness (self-reported WP, maximum handgrip strength [HGM], and RHR) can be used to improve or replace traditional risk factors in predicting all-cause mortality in both healthy populations and those living with existing health conditions.

## Patients and Methods

### UK Biobank

This analysis used data from UK Biobank (application #83285), a prospective cohort study of over 500,000 men and women aged 40-69 years,^31^ with data collected between 2006 and 2010 across 22 study assessment centers throughout England, Scotland, and Wales. Ethical approval for UK Biobank was obtained from the North West Centre for Research Ethics Committee (MREC, 11/NW/0382). In Scotland, UK Biobank has approval from the Community Health Index Advisory Group (CHIAG). Written informed consent was obtained from all participants before participation.

### Cohort Definition

**Figure 1:**
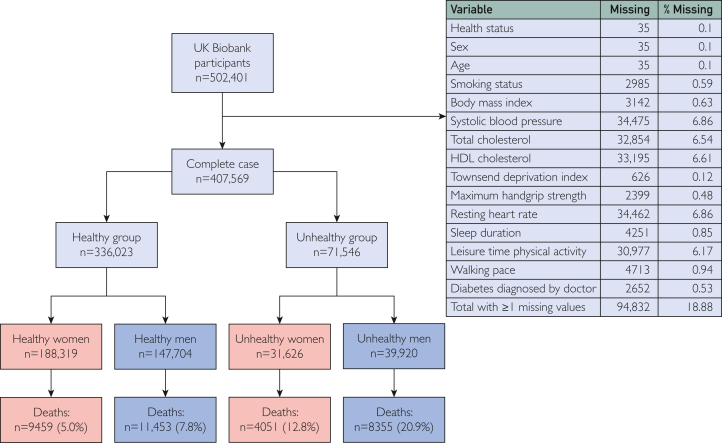
Flow chart. HDL, high-density lipoprotein.

From the initial sample of 502,401 participants (participants were recruited from May 1, 2006, and were followed until September 30, 2022), we excluded those with missing values in measures of physical behavior, function, and fitness and model covariates, leaving 407,569 participants for analysis (Figure 1). Our cohort was stratified into 4 subgroups based on presence of a health conditions and sex: healthy women, unhealthy women, healthy men, and unhealthy men. Participants with at least 1 prevalent illness from a list of 131 diseases at baseline, which reflect conditions commonly applied in clinical risk stratification or insurance underwriting (Supplemental Table 1, available online at http://www.mcpiqojournal.org), were classified as unhealthy.

### Physical Behavior, Function, and Fitness Risk Predictors

The 5 risk predictors of interest were self-reported or objectively assessed measures of physical behavior, function, and fitness: HGM, RHR, self-reported sleep duration, self-reported LTPA, and self-reported WP.

HGM (in kilograms) was assessed using a hydraulic hand dynamometer (Jamar J00105) while sitting. Both left- and right-hand grip strength (UK Biobank Data Field [DF] 46 and 47) were measured, and the peak of either recorded value was used. Further, RHR (DF 4194) was measured after participants had sat quietly for 15 minutes and recorded in beats per minute. Sleep duration (DF 1160) data were collected using a self-reported questionnaire, with responses ranging from 1 to 23 hours and measured in hours per day. Participants were asked, “How many hours sleep do you get in every 24 hours?”

Physical activity was specified as LTPA referring to activities undertaken during free time. It was assessed by self-reported questionnaire and participants were asked, “How many times in the last 4 weeks did you do: walking for pleasure (DF 971 for frequency; DF 981 for duration), heavy DIY (ie, do it yourself; DF 2624 for frequency; DF 2634 for duration), strenuous sports (DF 991 for frequency; DF 1001 for duration), and other exercises (DF 3637 for frequency; DF 3647 for duration)?”. Participants could select more than 1 activity and were asked to quantify their participation by frequency (ie, number of times in the previous 4 weeks) and duration. The intensity was expressed in terms of standardized metabolic equivalent of task (MET) values: 3.5 METs for walking for pleasure; 8.0 METs for strenuous sports; 4.0 METs for other activities. The total weekly LTPA (MET-minutes/week) was calculated by multiplying the frequency, duration, and assigned MET values as reported previously.^32^

Waking pace (DF 924) was assessed by self-reported questionnaire, and participants were asked to answer the following question: “How would you describe your usual WP: (1) slow pace, (2) steady/average WP, and (3) brisk pace?”

### Traditional Risk Factors

Data for traditional risk factors was also included as follows: age (years); smoking status (never, previous, and current); BMI (in kg/m^2^); SBP (in mm Hg); total cholesterol-to–HDL cholesterol ratio (CHR; in mmol/L); and Townsend deprivation score. Age (DF 21022), Townsend deprivation index (DF 22189), smoking status (DF 20116), BMI (DF 21001), SBP (DF 4080), and CHR were collected at the recruitment (2006 to 2010). Moreover, CHR was calculated from measured total cholesterol (DF 30690) and HDL cholesterol (DF 30760). The Townsend deprivation score (DF 22189) is based upon participants home postcode at enrollment and is a composite of 4 domains: unemployment, household overcrowding, nonhome ownership, and noncar ownership. A lower score represents lower material deprivation.

A trained health care professional measured participants’ SBP, using an automated Omron HEM-705IT digital blood pressure monitor after participants had been seated at rest for at least 5 minutes, and took blood samples at their assessment center visits. Total cholesterol and HDL cholesterol in serum samples were measured using the Beckman Coulter AU5800 analytical platform, and analysis method was by enzyme immunoinhibition and CHO-POD analysis, respectively.

### Outcome

All-cause mortality was used as the outcome for this analysis. Linked National Health Service (NHS; England and Wales) and NHS Central Register (Scotland) data provided information on date and cause of death. Follow-up time started at the baseline assessment center visit and terminated at the earliest occurrence of death or censoring (November 30, 2022, for England, Wales, and Scotland).

### Statistical Analyses

Descriptive statistics were undertaken to summarize the baseline characteristics of the study population, including demographic, clinical and physical behavior, fitness, and function factors.

The analysis was carried out in 2 phases to address the aim of the study. Both phases used a base model of traditional risk factors as follows: age, smoking status, BMI, SBP, CHR, and Townsend deprivation.

All continuous base model covariates (ie, age, BMI, SBP, CHR, and deprivation) and measures of physical behavior, function, and fitness (ie, RHR, HGM, sleep, and LTPA) were fitted with spline terms where appropriate. For each of these variables, we compared an individually adjusted model with a linear vs nonlinear (spline) transformation of the variable, with the number of knots ranging from 3 to 6 located at centiles of the variable, as suggested by Harrell and Frank.^33^ We assessed the Bayesian Information Criterion for each model (linear vs nonlinear) and number of knots. The model with the lowest Bayesian Information Criterion was chosen for each covariable and risk predictor. Knots were capped at 6 to avoid overfitting but limited to 3 for the sleep variable owing to its more discrete distribution. All continuous variables were centered at the median value with respect to stratifications by sex and health conditions, regardless of whether being fitted with a spline or not. All categorical variables—WP and smoking status—were fitted with their original terms.

In phase 1, Royston–Parmar flexible parametric survival models were applied to estimate the hazard ratios (HRs) and their corresponding 95% CI for the 5 measures of physical behavior and markers of physical function and fitness (HGM, RHR, sleep duration, LTPA, and WP). Six models were developed by adding these measures to the base model, both individually and combined.

In phase 2, Harrell concordance index (C-index) and net reclassification indices (NRIs) were estimated after applying a Cox proportional hazard model to evaluate the performance of the prediction models. Three steps were undertaken.

In step 1, the model performance was evaluated when the 5 measures of interest were added to the base model, either individually or combined, to determine whether their inclusion improved the prognostic discrimination of the base model. In step 2, CHR or SBP in the base model were replaced by the 5 measures of interest, either individually or combined, to evaluate whether easy-to-collect measures of physical behavior, function, and fitness factors can be substituted for traditional clinical risk factors and still provide similar prognostic discrimination. Step 3 substituted both CHR and SBP at the same time.

For model interpretation, a C-index value of <0.6 was considered poor predictive discrimination, 0.6-0.7 as moderate predictive discrimination, and >0.7 as good predictive discrimination.^34^ To evaluate reclassification performance, the NRI was calculated at the 10-year mortality risk thresholds of 5%, 7.5%, and 10%.^35^

All analyses were stratified by sex and health status and carried out on complete case data. All analyses were conducted using the Stata/BE (version 18.0; STATA) and R software (version 4.3.1; R Foundation for Statistical Computing).

### Sensitivity Analysis

In order to assess whether the model performance for those in the unhealthy group was consistent when considering major prevalent diseases separately, the analysis was repeated in those with prevalent CVD and prevalent cancer. In addition, to minimize the potential for reverse causality, we conducted a further sensitivity analysis excluding participants who died within the first 2 years of follow-up. This approach resulted a cohort of 405,781 participants prior to any stratifications.

## Results

### Population Characteristics

The Table presents the characteristics of the 407,569 participants, stratified by sex and health status—healthy women (n=188,319 [46.2%]; 9459 [5.0%] deaths during the follow-up), unhealthy women (n=31,626 [7.8%]; 4051 [12.8%] deaths), healthy men (n=147,704 [36.2%]; 11,453 [7.8%] deaths), and unhealthy men (n=39,920 [9.8%]; 8355 [20.9%] deaths). Each stratified group had a median follow-up time of 13.5, 13.2, 13.4, and 12.7 years, respectively; survival curves for each cohort are displayed in Supplemental Figure 1 (available online at http://www.mcpiqojournal.org). Women and men in the unhealthy groups had lower levels of HGM and physical activity, higher RHR values, and a higher proportion of slow walkers. The unhealthy group also had a lower lower-quantile value for sleep, ranging from 6 hours despite the shared upper limit of 8 hours with the healthy group (Table).

**Table:** Characteristics of Stratified Groups, by Sex and Health Status

Characteristic	Women		Men		Total
	Healthy	Unhealthy	Healthy	Unhealthy	
Participants	188,319 (46.2)	31,626 (7.8)	147,704 (36.2)	39,920 (9.8)	407,569
Deaths	9459 (5.0)	4051 (12.8)	11,453 (7.8)	8355 (20.9)	33,318 (8.2)
Age at recruitment (y)	57 (49-62)	60 (54-65)	57 (49-63)	62 (56-66)	58 (50-63)
Systolic blood pressure (mm Hg)	135 (122-149)	137 (125-151)	141 (130-154)	141 (129-154)	138 (126-152)
Smoking status					
Never	114,647 (60.9)	16,972 (53.7)	77,019 (52.1)	15,581 (39.0)	224,219 (55.0)
Previous	58,537 (31.1)	11,212 (35.5)	53,594 (36.3)	19,073 (47.8)	142,416 (34.9)
Current	15,135 (8.0)	3442 (10.9)	17,091 (11.6)	5266 (13.2)	40,934 (10.0)
Cholesterol (mmol/L)	5.9 (5.2-6.6)	5.4 (4.6-6.2)	5.6 (5.0-6.3)	4.7 (4.0-5.5)	5.7 (4.9-6.4)
HDL cholesterol (mmol/L)	1.6 (1.4-1.9)	1.5 (1.2-1.8)	1.3 (1.1-1.5)	1.2 (1.0-1.4)	1.4 (1.2-1.7)
Cholesterol-to-HDL-cholesterol ratio	3.6 (3.0-4.3)	3.6 (3.0-4.3)	4.3 (3.6-5.2)	3.9 (3.3-4.7)	3.9 (3.2-4.7)
Body mass index (kg/m^2^)	25.8 (23.3-29.1)	27.5 (24.3-31.8)	27.0 (24.8-29.6)	28.2 (25.6-31.3)	26.6 (24.1-29.7)
Townsend deprivation index at recruitment	−2.3 (−3.7 to 0.2)	−1.7 (−3.4 to 1.3)	−2.3 (−3.7 to 0.2)	−1.8 (−3.5 to 1.3)	−2.2 (−3.7 to 0.4)
Handgrip strength (kg)	26.0 (22.0-30.0)	23.0 (19.0-28.0)	42.0 (36.0-48.0)	40.0 (34.0-46.0)	31.0 (24.0-40.0)
Resting heart rate (beats/min)	69 (63-76)	71 (63-79)	67 (60-75)	68 (60-78)	68 (61-76)
Usual walking pace					
Slow pace	9590 (5.1)	6201 (19.6)	5987 (4.1)	6826 (17.1)	28,604 (7.0)
Steady average pace	99,082 (52.6)	17,024 (53.8)	76,223 (51.6)	22,035 (55.2)	214,364 (52.6)
Brisk pace	79,647 (42.3)	8401 (26.6)	65,494 (44.3)	11,059 (27.7)	164,601 (40.4)
Sleep duration (h)	7 (7-8)	7 (6-8)	7 (7-8)	7 (6-8)	7 (7-8)
Leisure time physical activity (MET-min/wk)	515.6 (182.8-1089.4)	371.2 (92.8-895.3)	732.2 (270.0-1617.0)	550.7 (154.7-1301.2)	576.8 (185.6-1251.1)

Values represent median (interquartile range) for continuous variables and frequency (%) for categorical variables. A smaller value of Townsend score indicates for lower levels of deprivation.

### Associations With Mortality

**Figure 2:**
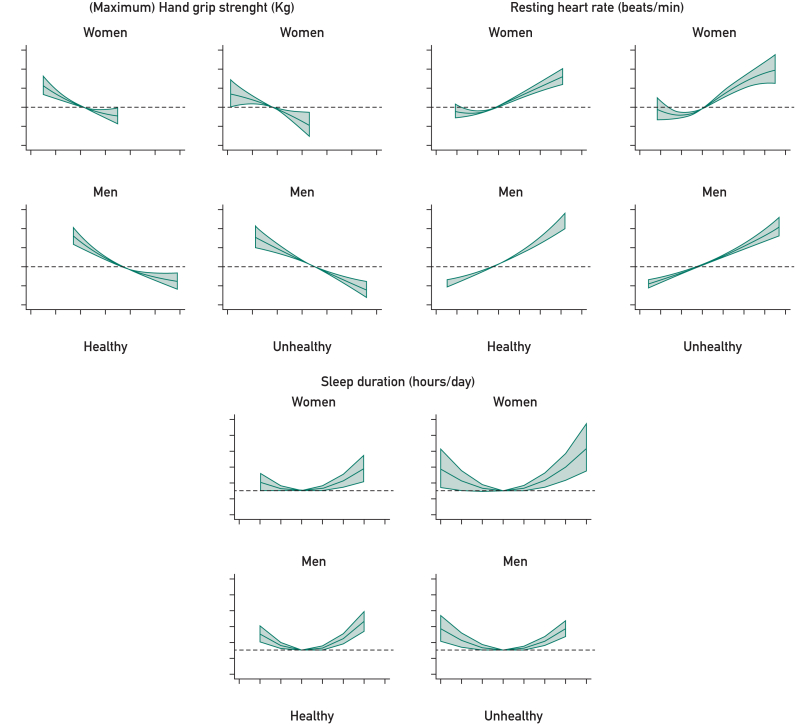

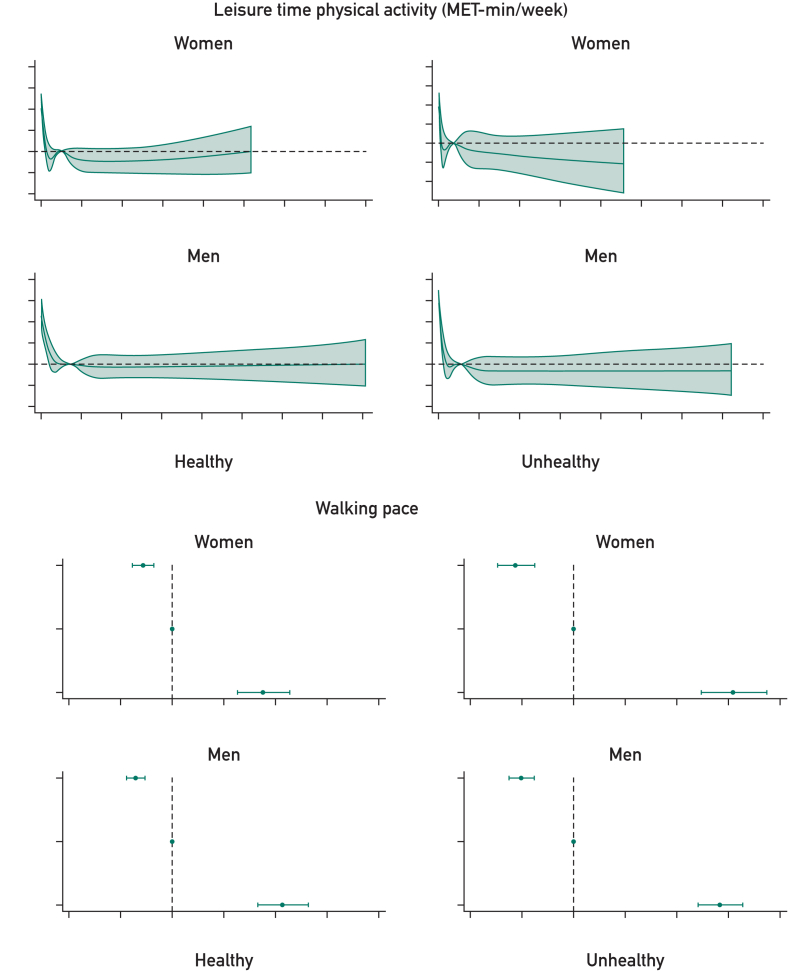
Association of measures of physical behaviors, function, and fitness with all-cause mortality. Each of the 5 subplots has 4 panels, stratified by sex and health status. The *x*-axis in panels of continues variables (ie, HGM, resting heart rate, sleep durations, and leisure time physical activity) represents the observed values of the variable. The *y*-axis represents the estimated hazard ratio (HR) derived from the mutually adjusted flexible parametric model (base model: age, smoking status, body mass index, systolic blood pressure, total cholesterol-to–high-density lipoprotein ratio, and deprivation). The solid line represents the estimated HR across the distribution of the variable, and the shaded area represents the 95% CI. All plots of continuous exposures are based on the 1st to 99th percentiles of the data distribution. For the forest plot panel, the *x*-axis represents for the HR and the *y*-axis, the 3 levels of the walking pace variable. The solid point represents for the estimated HR and the error bar, the 95% CI.

Figure 2 shows the HRs for the 5 measures of physical behavior, function, and fitness when added to the base model as a combination. The pattern of association for HGM, RHR, sleep duration, and LTPA with all-cause mortality was similar across all 4 subgroups. Moreover, HGM reported an inverse association with mortality, whereas RHR was positively associated, with an approximately linear association in both healthy and unhealthy groups. Sleep duration showed a U-shaped relationship across all subgroups, with both shorter and longer sleep duration associated with higher mortality risk than the reference median of 7 hours. For WP, slow walkers were found to have higher HRs and brisk walkers lower HRs across all groups, than the average walkers. More pronounced HRs were observed for slow walkers in the unhealthy population (women: 1.62; 95% CI, 1.50-1.75; men: 1.56; 95% CI, 1.48-1.65) than those with the healthy population (women: 1.36; 95% CI, 1.26-1.46; men: 1.43; 95% CI, 1.33-1.53).

### Prognostic Discrimination

#### Step 1

**Figure 3:**
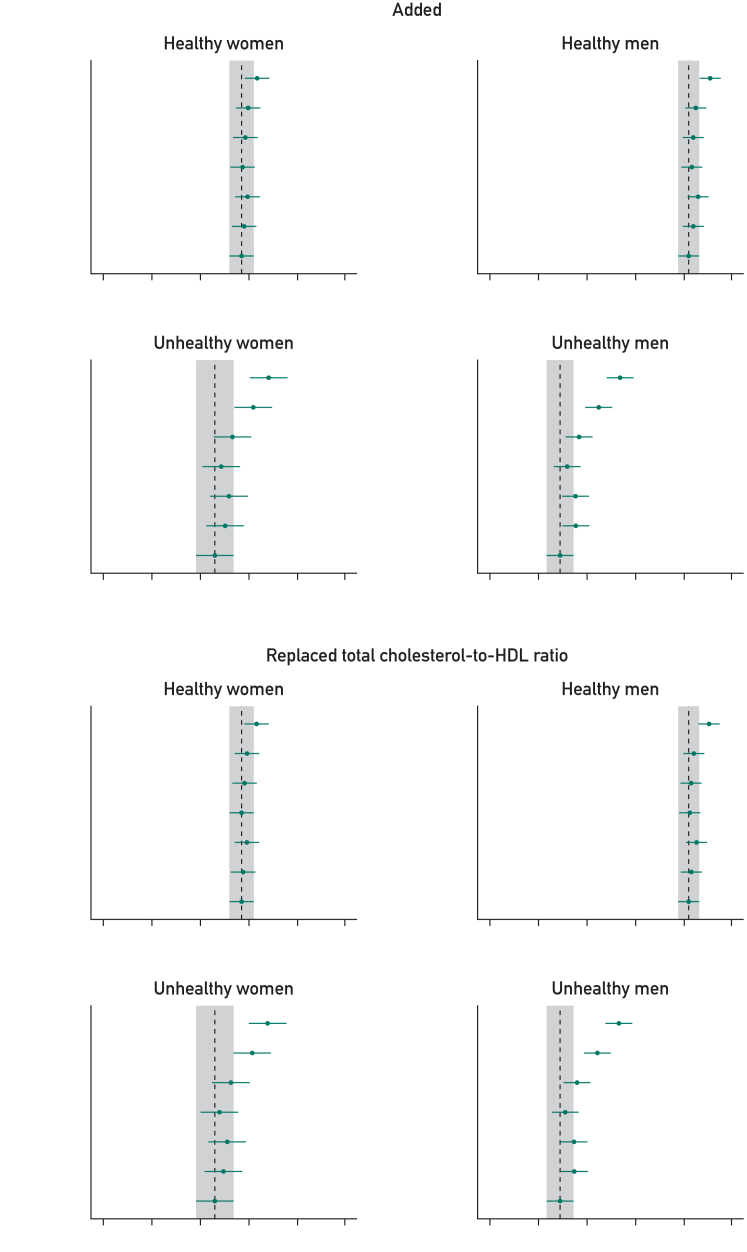

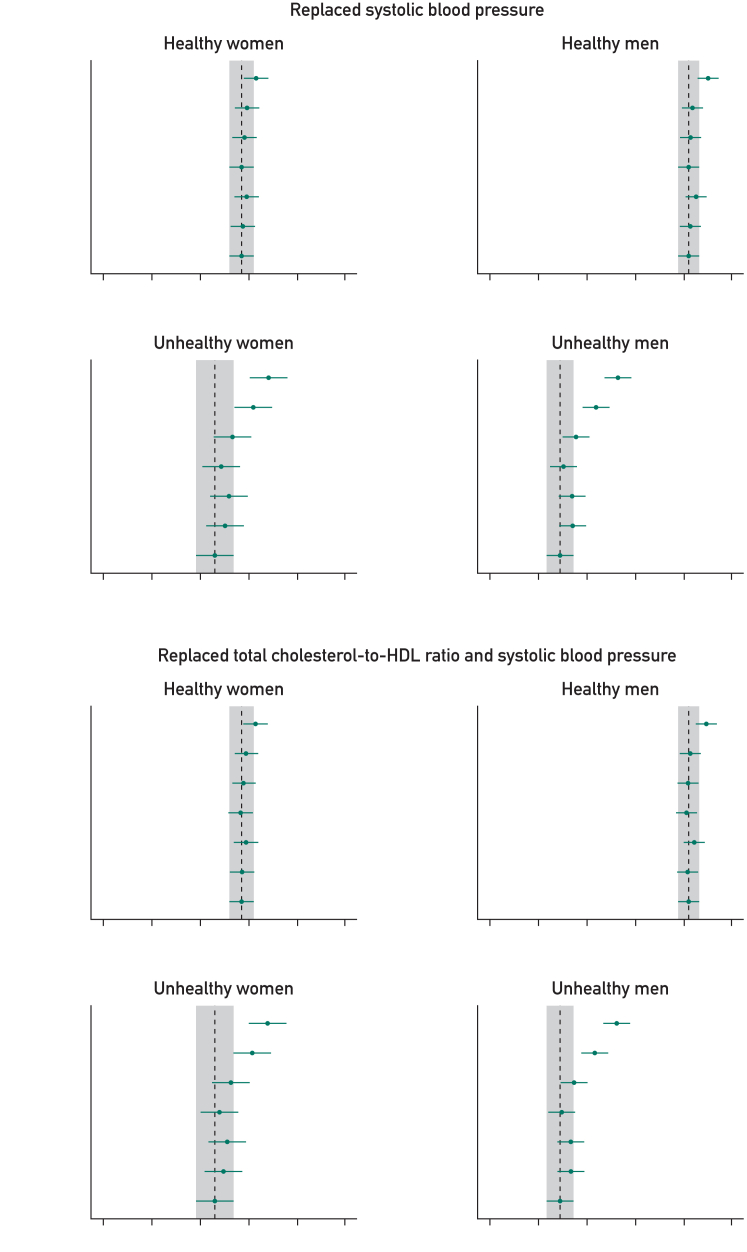
C-index comparing base models vs new models with added or replaced physical behavior, function, and fitness measures, stratified by health status and sex. All represents base model covariates and 5 physical behavior, fitness, and function indicators (WP, LTPA, RHR, HGM, and sleep). The top-left panel represents for the addition step and the remaining 3 panels the substitution steps (replacing SBP, CHR, or both). Within each panel, plots display results stratified by sex and health conditions. The *x*-axis shows the range of C-index values, while the *y*-axis lists the variables considered at each step. In each plot, the point estimate represents for the predicted C-index for the modified models and the error bar, the corresponding 95% CI. The base model serves as the reference (age, smoking status, BMI, SBP, CHR, and deprivation). The dotted gray line represents the C-index of the base model, with the gray shaded area, the 95% CI. BMI, body mass index; CHR, total cholesterol-to-HDL-cholesterol ratio; HGM, handgrip strength (maximum); LTPA, leisure time physical activity; RHR, resting heart rate; SBP, systolic blood pressure; WP, walking pace.

In the healthy cohort, the base model (age, smoking status, BMI, SBP, CHR, and Townsend deprivation) provided good prognostic discrimination in women (0.707; 95% CI, 0.702-0.712) and men (0.732; 95% CI, 0.728-0.737). The addition of individual or combined measures of physical behavior, function, and fitness to the base model provided small improvements to prognostic discrimination, with the combination of all 5 predictors providing the greatest difference (difference in C-index for women: 0.006 [95% CI, 0.005-0.008] and for men: 0.009 [95% CI, 0.008-0.010]) (Figure 3; Supplemental Table 2, available online at http://www.mcpiqojournal.org). However, risk classification was not meaningfully affected (Supplemental Table 6, available online at http://www.mcpiqojournal.org).

For the unhealthy cohort, the base model provided moderate prognostic discrimination in women (0.696; 95% CI, 0.688-0.704) and men (0.679; 95% CI, 0.674-0.685). However, the addition of the combined measures of physical behavior, function, and fitness improved the model to achieve good prognostic discrimination in women (0.719; 95% CI, 0.711-0.726; difference: 0.022; 95% CI, 0.018-0.026) and men (0.704; 95% CI, 0.698-0.710; difference: 0.025; 95% CI, 0.022-0.028) (Figure 3; Supplemental Table 2). Risk classification improved in the unhealthy cohort. The addition of the combined physical measures lead to an overall net reclassification for the 10-year mortality risk prediction observed in 10.2% (95% CI, 7.1%-13.3%) of women and 20.2% (95% CI, 17.9%-22.5%) of men (Supplemental Table 6).

Individually, WP was the most prognostically powerful individual risk factor in women and men in the unhealthy cohort, significantly improving the C-index to an order of magnitude greater than other risk factors, whereas improving the risk reclassification to a similar level as all risk factors combined (Figure 3; Supplemental Tables 2 and 6).

#### Steps 2 and 3

In all cohorts, replacing SBP, CHR, or both with measures of physical behavior, function, and fitness lead to a small gains in prognostic discrimination in most models, with more notable and consistent differences observed in unhealthy women and men (Figure 3; Supplemental Tables 3-5, available online at http://www.mcpiqojournal.org). For example, in the model replacing both SBP and CHR with all considered measures of physical behavior, function, and fitness, the C-index improved by 0.006 (95% CI, 0.004-0.007) in healthy women and 0.007 (95% CI, 0.006-0.009) in healthy men, with larger improvements of 0.022 (95% CI, 0.018-0.026) observed for unhealthy women and 0.023 (95% CI, 0.020-0.027) in unhealthy men. Risk classification (NRI) was only significantly improved in the unhealthy cohorts (women: 9.6% [95% CI, 6.5%-12.8%]; men: (19.0% [95% CI, 16.6%-21.4%]) (Supplemental Table 6).

Walking pace was consistently the best performing single risk factor in substitution models. For example, substituting WP for both SBP and CHR improved both prognostic discrimination and risk classification in both women and men (Figure 3; Supplemental Tables 3-5), with the C-index improving by 0.015 (95% CI, 0.012-0.019) and an NRI of 11.0% (95% CI, 7.4%-14.6%) in women. In men, results are more pronounced with improvements of 0.014 (95% CI, 0.012-0.017) in C-index and an NRI of 14.0% (95% CI, 11.5%-16.5%) (Figure 3; Supplemental Tables 5 and 6).

### Sensitivity Analysis

Similar patterns were observed for the unhealthy group when focusing only on prevalent CVD or cancer rather than the wider list of health conditions used in the main analysis. In those with either prevent disease, combined measures of physical behavior, function, and fitness significantly improved prognostic discrimination of the models, particularly in men with prevalent CVD, although with wider CIs. When considering measures of physical activity, function, and fitness individually, WP was also associated with the largest increases to the C-index (Supplemental Figure 2, available online at http://www.mcpiqojournal.org).

Trends in prognostic discrimination when excluding participants who died in the first 2 years follow-up were slightly attenuated, but the overall pattern and interpretation was consistent with the main analyses (Supplemental Figure 3, available online at http://www.mcpiqojournal.org).

## Discussion

Findings suggested that although incorporating combined measures of physical behaviors, fitness, and function into traditional risk models may improve predictive accuracy in all considered subgroups, results were the strongest and most consistent in women and men living with prevalent health conditions. In this population, prognostic discrimination and risk classification was also improved by substituting the traditional predictors of SBP and CHR with these combined indicators. Furthermore, among the 5 considered indicators, WP was the strongest individual indicator, significantly improving predictive performance and risk classification when used in place of SBP and CHR measures within the base model, particularly within the unhealthy population. For example, when both SBP and CHR were replaced with WP as a single factor, the net reclassification in risk resulted in 11.0% (95% CI, 7.4%-14.6%) of unhealthy women and 14.0% (95% CI, 11.5%-16.5%) of unhealthy men being assigned a more accurate 10-year risk category.

Measures of physical behavior, fitness, and function are established determinants of all-cause mortality, with attaining or maintaining healthy levels of these factors widely recommended for disease prevention.^18^^,^^36^^,^^37^ However, few studies have systematically investigated whether these simple to measure markers can be added or substituted for traditional clinical markers in mortality prediction. As far as we are aware, this is the first study to compare the predictive discrimination of physical behavior, fitness, and function indicators as additions to or substitutions for established risk predictors, and we observed substantial improvements in model performance when compared with previous literature. The observed C-index gain of 0.025 in our study is particularly noteworthy when compared with established prognostic markers in the existing literature. For instance, prior research showed a maximum C-index improvement of 0.022 for all-cause mortality when incorporating lifestyle factors,^23^ whereas the addition of C-reactive protein or polygenic risk scores often yields more modest gains of less than 0.015 and 0.01, respectively.^38^^,^^39^ Our results highlight potential to enhance the performance of risk predictions models, particularly in the situation where variables are collected in a nonclinical setting, such as public facing risk prediction calculators and where risk prediction is undertaken in nonclinical setting or sectors when data for risk factors such as cholesterol may not be readily available, particularly for populations with pre-existing health conditions. Furthermore, even within clinical setting, the addition of lifestyle factors into risk prediction models may add value. Importantly, traditional risk scores contain nonmodifiable risk factors or risk factors that are subject to medical management. Therefore, behavior change is not incentivized because it does not directly affect risk prediction. This limitation was recognized by the developers of the Finnish Diabetes Risk Score, which included questions related to diet and physical activity for educational purposes.^40^ We show that such additions also have the potential to improve risk prediction.

Along with the potential for combining measures of physical behavior, function, and fitness to improve prognostic discrimination, this study also reported that WP was the strongest individual predictor of mortality, particularly in those with a prevalent health condition. This finding is consistent with prior research, including a UK Biobank study that reported WP being one of the top predictors of mortality of 655 variables tested across sexes.^24^ Another study further evaluated its prognostic power by considering it alongside the established European SCORE^41^ model, with significant improvements in model performance (C-index) found by 0.008 in women and 0.013 in men.^23^ Our findings extend previous literatures by suggesting that WP not only performs as a stronger risk predictor but also outperforms traditional measures such as SBP or CHR in populations with prevalent health conditions. Interestingly, none of the other physical behavior, function, or fitness indicators improved prognostic discrimination when added individually.

The strong results for WP may reflect an ability to capture information reflecting underlying systems of physical robustness and homeostasis not captured by traditional clinical measures. For example, WP provides a robust measure of cardiorespiratory fitness,^21^ is a key indicator within frailty measures,^42^ and is commonly used as a primary outcome in rehabilitation studies through the application of walking tests.^43^ It is also shown to share a genetic overlap with multiple chronic conditions, along with behavioral and cognitive traits,^44^ supporting its role as a summary measure for the health of many different biological and behavioral pathways and as such, acts as a unique prognostic indicator. Given its simplicity and robust prognostic power,^23^^,^^45^ WP could be incorporated into public facing risk prediction calculators or potentially within electronic health records as a single self-reported item and implemented in risk models to improve the identification of individuals at risk. For example, infrastructure already exists through the NHS within England for classifying and coding physical activity levels through the GP Physical Activity Questionnaire.^46^ A single-item WP question is already embedded within GP Physical Activity Questionnaire could be added to this existing physical activity coding structure.

The strengths of the present study include the large sample size and number of outcomes, which enabled robust analyses. This extensively phenotyped cohort also provides detailed data on lifestyle and physical function measures that are not typically captured in electronic health records.

There are also important limitations. First, this analysis was not designed to develop a risk score but, rather, generate hypotheses and emphasize the potential prognostic relevance of measures of physical behavior, function, and fitness in future mortality prediction research. Second, physical health measures of sleep hours, LTPA, and WP were assessed using self-reported questionnaires, which may be prone to overestimation, misclassification, and reporting bias,^47^ which could weaken the observed prognostic discrimination.^48^ However, the prognostic strength of WP, despite the self-reported method used, may reflect the strength of association between self-reported WP and objectively assessed physical activity and cardiorespiratory fitness,^21^^,^^45^ along with causal associations reported for biological age and cardiovascular health,^44^^,^^49^ making it a validated novel marker of whole body health. Finally, UK Biobank is not representative of the general population, which may limit the generalizability of findings to other populations.^50^

## Conclusion

In conclusion, this study suggests the potential of including simple to measure physical behavior, function, and fitness indicators to improve the prediction of all-cause mortality risk beyond the typical established risk factors. These findings highlight 2 key points. First, self-reported WP showed particularly strong predictive discrimination across stratifications. Second, predictive discrimination for all-cause mortality was enhanced to a greater extent among populations living with a prevalent health condition. Future research should explore whether these findings have utility in improving existing risk scores.

## Potential Competing Interests

Dr Davies reports research grants from AstraZeneca, Boehringer Ingelheim, and Novo Nordisk; consulting fees from Boehringer Ingelheim, Lilly, Novo Nordisk, and Sanofi; honoraria from AstraZeneca, Boehringer Ingelheim, Novo Nordisk, Sanofi, Lilly, and Zuellig; travel support from Boehringer Ingelheim, Novo Nordisk, Lilly, Amgen, AstraZeneca, Biomea Fusion, Regeneron, and Zealand Pharma; and participation in data safety monitoring boards of Amgen, AstraZeneca, Biomea Fusion, Sanofi, Zealand Pharma, Carmot/Roche, Regeneron, EktaH, AbbVie, GSK, Daewoong Pharmaceuticals, and Innovent Bio. Dr Yates reports institutional grants from NIHR Leicester BRC and RGA: Reinsurance Group of America. The other authors report no competing interests.

## Ethics Statement

Ethical approval for UK Biobank was obtained from the North West Centre for Research Ethics Committee (MREC, 11/NW/0382). In Scotland, UK Biobank has approval from the Community Health Index Advisory Group (CHIAG). Written informed consent was obtained from all participants before participation.
