# Supplementary material for: The Utility of Measures of Physical Behavior, Function, and Fitness as Predictors of Mortality
Source: Mayo Clin Proc Innov Qual Outcomes. 2026 Mar 17;10(2):100710. doi: 10.1016/j.mayocpiqo.2026.100710 (PMC13010521; doi:10.1016/j.mayocpiqo.2026.100710)
Supplement: Supplementary Material [file mmc1.docx]

***SUPPLEMENTARY MATERIAL***

Table of content

[**Table S1**: Disease list. 1](#_Toc220230899)

[**Table S2:** Comparisons of C-index between base models and models after adding physical measures of behaviour, function, and fitness 4](#_Toc220230900)

[**Table S3:** Comparisons of C-index between base models and new models after replacing cholesterol-to-HDL ratio with physical measures of behaviour, function, and fitness 5](#_Toc220230901)

[**Table S4:** Comparisons of C-index between base models and new models after replacing systolic blood pressure with physical measures of behaviour, function, and fitness 7](#_Toc220230902)

[**Table S5:** Comparisons of C-index between base models and new models after replacing both cholesterol-to-HDL ratio and systolic blood pressure with physical measures of behaviour, function, and fitness 8](#_Toc220230903)

[**Table S6:** Net Reclassification Indices for 5-year survival probability prediction comparing the base model with the new model with physical behaviour, physical function, and fitness measures in each step. 9](#_Toc220230904)

[**Figure S1**: Kaplan–Meier survival curves comparing survival probability across stratification on sex and health conditions. 11](#_Toc220230905)

[**Figure S2**: Sensitivity analyses on C-index in people with CVD and cancer comparing base models versus models with added or substituted physical behaviour, function, and fitness measures 12](#_Toc220230906)

[**Figure S3:** Sensitivity analyses of the C-index in participants excluding those who died within the first two years, comparing base models with models that included or substituted physical behaviour, function, and fitness measures. 14](#_Toc220230907)

Table S1: Disease list.

|  | Coding | Definition |
| --- | --- | --- |
| 1 | 1066 | heart/cardiac problem |
| 2 | 1067 | peripheral vascular disease |
| 3 | 1068 | venous thromboembolic disease |
| 4 | 1074 | angina |
| 5 | 1075 | heart attack/myocardial infarction |
| 6 | 1076 | heart failure/pulmonary odema |
| 7 | 1077 | heart arrhythmia |
| 8 | 1078 | heart valve problem/heart murmur |
| 9 | 1079 | cardiomyopathy |
| 10 | 1081 | stroke |
| 11 | 1082 | transient ischaemic attack (tia) |
| 12 | 1083 | subdural haemorrhage/haematoma |
| 13 | 1086 | subarachnoid haemorrhage |
| 14 | 1088 | arterial embolism |
| 15 | 1112 | chronic obstructive airways disease/copd |
| 16 | 1113 | emphysema/chronic bronchitis |
| 17 | 1115 | interstitial lung disease |
| 18 | 1120 | asbestosis |
| 19 | 1121 | pulmonary fibrosis |
| 20 | 1122 | fibrosing alveolitis/unspecified alveolitis |
| 21 | 1124 | respiratory failure |
| 22 | 1136 | liver/biliary/pancreas problem |
| 23 | 1141 | oesophageal varicies |
| 24 | 1155 | hepatitis |
| 25 | 1157 | non-infective hepatitis |
| 26 | 1158 | liver failure/cirrhosis |
| 27 | 1164 | pancreatic disease |
| 28 | 1165 | pancreatitis |
| 29 | 1191 | gastrointestinal bleeding |
| 30 | 1192 | renal/kidney failure |
| 31 | 1193 | renal failure requiring dialysis |
| 32 | 1194 | renal failure not requiring dialysis |
| 33 | 1220 | diabetes |
| 34 | 1222 | type 1 diabetes |
| 35 | 1223 | type 2 diabetes |
| 36 | 1233 | adrenal tumour |
| 37 | 1236 | phaeochromocytoma |
| 38 | 1238 | pituitary adenoma/tumour |
| 39 | 1240 | neurological injury/trauma |
| 40 | 1244 | infection of nervous system |
| 41 | 1251 | spinal cord disorder |
| 42 | 1252 | paraplegia |
| 43 | 1258 | chronic/degenerative neurological problem |
| 44 | 1259 | motor neurone disease |
| 45 | 1260 | myasthenia gravis |
| 46 | 1261 | multiple sclerosis |
| 47 | 1262 | parkinsons disease |
| 48 | 1263 | dementia/alzheimers/cognitive impairment |
| 49 | 1267 | spinal injury |
| 50 | 1276 | diabetic eye disease |
| 51 | 1289 | schizophrenia |
| 52 | 1290 | deliberate self-harm/suicide attempt |
| 53 | 1291 | mania/bipolar disorder/manic depression |
| 54 | 1327 | low platelets/platelet disorder |
| 55 | 1328 | haemophilia |
| 56 | 1331 | pernicious anaemia |
| 57 | 1332 | aplastic anaemia |
| 58 | 1339 | sickle cell disease |
| 59 | 1371 | sarcoidosis |
| 60 | 1372 | vasculitis |
| 61 | 1373 | connective tissue disorder |
| 62 | 1376 | giant cell/temporal arteritis |
| 63 | 1378 | wegners granulmatosis |
| 64 | 1379 | microscopic polyarteritis |
| 65 | 1380 | polyartertis nodosa |
| 66 | 1381 | systemic lupus erythematosis/sle |
| 67 | 1383 | dermatopolymyositis |
| 68 | 1384 | scleroderma/systemic sclerosis |
| 69 | 1397 | other demyelinating disease (not multiple sclerosis) |
| 70 | 1408 | alcohol dependency |
| 71 | 1409 | opioid dependency |
| 72 | 1410 | other substance abuse/dependency |
| 73 | 1425 | cerebral aneurysm |
| 74 | 1426 | myocarditis |
| 75 | 1427 | polycystic kidney |
| 76 | 1432 | carcinoid syndrome/tumour |
| 77 | 1433 | cerebral palsy |
| 78 | 1434 | other neurological problem |
| 79 | 1435 | optic neuritis |
| 80 | 1437 | myasthenia gravis |
| 81 | 1438 | polycythaemia vera |
| 82 | 1439 | hiv/aids |
| 83 | 1445 | clotting disorder/excessive bleeding |
| 84 | 1447 | pancytopenia |
| 85 | 1448 | neutropenia/lymphopenia |
| 86 | 1449 | myeloproliferative disorder |
| 87 | 1450 | monoclonal gammopathy/not myeloma |
| 88 | 1451 | hereditary/genetic haematological disorder |
| 89 | 1462 | crohns disease |
| 90 | 1463 | ulcerative colitis |
| 91 | 1464 | rheumatoid arthritis |
| 92 | 1468 | diabetic neuropathy/ulcers |
| 93 | 1470 | anorexia/bulimia/other eating disorder |
| 94 | 1471 | atrial fibrillation |
| 95 | 1472 | emphysema |
| 96 | 1475 | sclerosing cholangitis |
| 97 | 1480 | dermatomyositis |
| 98 | 1481 | polymyositis |
| 99 | 1483 | atrial flutter |
| 100 | 1486 | sick sinus syndrome |
| 101 | 1489 | mitral stenosis |
| 102 | 1490 | aortic stenosis |
| 103 | 1491 | brain haemorrhage |
| 104 | 1492 | aortic aneurysm |
| 105 | 1493 | other venous/lymphatic disease |
| 106 | 1496 | alpha-1 antitrypsin deficiency |
| 107 | 1506 | primary biliary cirrhosis |
| 108 | 1507 | haemochromatosis |
| 109 | 1519 | kidney nephropathy |
| 110 | 1520 | iga nephropathy |
| 111 | 1521 | diabetes insipidus |
| 112 | 1524 | spina bifida |
| 113 | 1526 | polio / poliomyelitis |
| 114 | 1546 | essential thrombocytosis |
| 115 | 1579 | hepatitis b |
| 116 | 1580 | hepatitis c |
| 117 | 1582 | hepatitis e |
| 118 | 1583 | ischaemic stroke |
| 119 | 1584 | mitral valve disease |
| 120 | 1585 | mitral regurgitation / incompetence |
| 121 | 1586 | aortic valve disease |
| 122 | 1587 | aortic regurgitation / incompetence |
| 123 | 1588 | hypertrophic cardiomyopathy (hcm / hocm) |
| 124 | 1591 | aortic aneurysm rupture |
| 125 | 1592 | aortic dissection |
| 126 | 1595 | pleural plaques (not known asbestosis) |
| 127 | 1601 | bowel / intestinal infarction |
| 128 | 1604 | alcoholic liver disease / alcoholic cirrhosis |
| 129 | 1607 | diabetic nephropathy |
| 130 | 1609 | glomerulnephritis |
| 131 | 1658 | myelofibrosis |

**Table S2:** Comparisons of C-index between base models and models after adding physical measures of behaviour, function, and fitness

|  |  | **Women** | | **Men** | |
| --- | --- | --- | --- | --- | --- |
| **Physical measure** | **Model** | **Healthy** | **Unhealthy** | **Healthy** | **Unhealthy** |
| Maximum  handgrip  strength | Base | 0.7073 (0.7020, 0.7125) | 0.6963 (0.6884, 0.7042) | 0.7324 (0.7279, 0.7369) | 0.6792 (0.6736, 0.6849) |
|  | New | 0.7084 (0.7031, 0.7136) | 0.7005 (0.6926, 0.7083) | 0.7344 (0.7299, 0.7389) | 0.6857 (0.6801, 0.6913) |
|  | **Difference** | 0.0011 (0.0005, 0.0017) | 0.0041 (0.0022, 0.0061) | 0.0020 (0.0013, 0.0027) | 0.0065 (0.0049, 0.0081) |
| Resting  heart  rate | Base | 0.7073 (0.7020, 0.7125) | 0.6963 (0.6884, 0.7042) | 0.7324 (0.7279, 0.7369) | 0.6792 (0.6736, 0.6849) |
|  | New | 0.7098 (0.7045, 0.7150) | 0.7021 (0.6943, 0.7100) | 0.7363 (0.7319, 0.7408) | 0.6856 (0.6799, 0.6913) |
|  | **Difference** | 0.0025 (0.0017, 0.0033) | 0.0058 (0.0036, 0.0080) | 0.0040 (0.0030, 0.0049) | 0.0064 (0.0046, 0.0082) |
| Sleep | Base | 0.7073 (0.7020, 0.7125) | 0.6963 (0.6884, 0.7042) | 0.7324 (0.7279, 0.7369) | 0.6792 (0.6736, 0.6849) |
|  | New | 0.7077 (0.7025, 0.7130) | 0.6991 (0.6914, 0.7069) | 0.7337 (0.7292, 0.7382) | 0.6825 (0.6769, 0.6881) |
|  | **Difference** | 0.0005 (0.0001, 0.0009) | 0.0028 (0.0012, 0.0044) | 0.0013 (0.0008, 0.0018) | 0.0033 (0.0021, 0.0044) |
| Leisure  time  physical  activity | Base | 0.7073 (0.7020, 0.7125) | 0.6963 (0.6884, 0.7042) | 0.7324 (0.7279, 0.7369) | 0.6792 (0.6736, 0.6849) |
|  | New | 0.7089 (0.7037, 0.7142) | 0.7036 (0.6957, 0.7114) | 0.7343 (0.7298, 0.7388) | 0.6870 (0.6814, 0.6926) |
|  | **Difference** | 0.0016 (0.0009, 0.0024) | 0.0073 (0.0048, 0.0097) | 0.0019 (0.0013, 0.0026) | 0.0078 (0.0060, 0.0095) |
| Walking  pace | Base | 0.7073 (0.7020, 0.7125) | 0.6963 (0.6884, 0.7042) | 0.7324 (0.7279, 0.7369) | 0.6792 (0.6736, 0.6849) |
|  | New | 0.7100 (0.7047, 0.7152) | 0.7122 (0.7044, 0.7199) | 0.7353 (0.7308, 0.7398) | 0.6952 (0.6896, 0.7008) |
|  | **Difference** | 0.0027 (0.0018, 0.0036) | 0.0158 (0.0124, 0.0193) | 0.0029 (0.0021, 0.0037) | 0.0160 (0.0134, 0.0185) |
| All physical measures | Base | 0.7073 (0.7020, 0.7125) | 0.6963 (0.6884, 0.7042) | 0.7324 (0.7279, 0.7369) | 0.6792 (0.6736, 0.6849) |
|  | New | 0.7137 (0.7085, 0.7189) | 0.7186 (0.7110, 0.7263) | 0.7413 (0.7368, 0.7458) | 0.7041 (0.6984, 0.7097) |
|  | **Difference** | 0.0064 (0.0050, 0.0078) | 0.0223 (0.0182, 0.0264) | 0.0089 (0.0075, 0.0104) | 0.0248 (0.0218, 0.0279) |

Base: C-index and 95%CI for base model (age, smoking status, body mass index (BMI), systolic blood pressure (SBP), total cholesterol-to-HDL ratio (CHR), and deprivation)

New: C-index and 95%CI for new model after adding either individual or all physical measures of behaviour, function, and fitness.

Difference: C-index and 95%CI for differences of C-index between base and new model.

Note: 95% confidence intervals for the difference in C-indices were calculated using non-parametric bootstrapping with 1,000 resamples.

**Table S3:** Comparisons of C-index between base models and new models after replacing cholesterol-to-HDL ratio with physical measures of behaviour, function, and fitness

|  |  | **Women** | | **Men** | |
| --- | --- | --- | --- | --- | --- |
| **Physical measure** | **Model** | **Healthy** | **Unhealthy** | **Healthy** | **Unhealthy** |
| Maximum  handgrip  strength | Base | 0.7073 (0.7020, 0.7125) | 0.6963 (0.6884, 0.7042) | 0.7324 (0.7279, 0.7369) | 0.6792 (0.6736, 0.6849) |
|  | New | 0.7080 (0.7027, 0.7132) | 0.6998 (0.6919, 0.7076) | 0.7335 (0.7290, 0.7380) | 0.6851 (0.6795, 0.6907) |
|  | **Difference** | 0.0007 (-0.0000, 0.0014) | 0.0035 (0.0014, 0.0055) | 0.0011 (0.0003, 0.0020) | 0.0059 (0.0042, 0.0076) |
| Resting  heart  rate | Base | 0.7073 (0.7020, 0.7125) | 0.6963 (0.6884, 0.7042) | 0.7324 (0.7279, 0.7369) | 0.6792 (0.6736, 0.6849) |
|  | New | 0.7095 (0.7043, 0.7147) | 0.7014 (0.6935, 0.7092) | 0.7356 (0.7312, 0.7401) | 0.6850 (0.6793, 0.6907) |
|  | **Difference** | 0.0022 (0.0013, 0.0031) | 0.0051 (0.0028, 0.0074) | 0.0033 (0.0022, 0.0043) | 0.0058 (0.0039, 0.0076) |
| Sleep | Base | 0.7073 (0.7020, 0.7125) | 0.6963 (0.6884, 0.7042) | 0.7324 (0.7279, 0.7369) | 0.6792 (0.6736, 0.6849) |
|  | New | 0.7073 (0.7021, 0.7125) | 0.6985 (0.6907, 0.7062) | 0.7328 (0.7283, 0.7373) | 0.6817 (0.6761, 0.6874) |
|  | **Difference** | 0.0001 (-0.0005, 0.0006) | 0.0021 (0.0004, 0.0039) | 0.0004 (-0.0003, 0.0011) | 0.0025 (0.0012, 0.0038) |
| Leisure  time  physical  activity | Base | 0.7073 (0.7020, 0.7125) | 0.6963 (0.6884, 0.7042) | 0.7324 (0.7279, 0.7369) | 0.6792 (0.6736, 0.6849) |
|  | New | 0.7086 (0.7033, 0.7138) | 0.7029 (0.6950, 0.7108) | 0.7334 (0.7289, 0.7379) | 0.6862 (0.6805, 0.6918) |
|  | **Difference** | 0.0013 (0.0005, 0.0021) | 0.0066 (0.0040, 0.0091) | 0.0010 (0.0002, 0.0019) | 0.0069 (0.0050, 0.0088) |
| Walking  pace | Base | 0.7073 (0.7020, 0.7125) | 0.6963 (0.6884, 0.7042) | 0.7324 (0.7279, 0.7369) | 0.6792 (0.6736, 0.6849) |
|  | New | 0.7096 (0.7044, 0.7149) | 0.7117 (0.7039, 0.7195) | 0.7345 (0.7300, 0.7390) | 0.6946 (0.6889, 0.7002) |
|  | **Difference** | 0.0023 (0.0014, 0.0033) | 0.0154 (0.0118, 0.0189) | 0.0021 (0.0012, 0.0031) | 0.0154 (0.0127, 0.0180) |
| All physical measures | Base | 0.7073 (0.7020, 0.7125) | 0.6963 (0.6884, 0.7042) | 0.7324 (0.7279, 0.7369) | 0.6792 (0.6736, 0.6849) |
|  | New | 0.7135 (0.7083, 0.7187) | 0.7182 (0.7105, 0.7259) | 0.7408 (0.7363, 0.7453) | 0.7035 (0.6979, 0.7092) |
|  | **Difference** | 0.0062 (0.0048, 0.0076) | 0.0219 (0.0177, 0.0260) | 0.0084 (0.0069, 0.0100) | 0.0243 (0.0212, 0.0274) |

Base: C-index and 95%CI for base model (age, smoking status, body mass index (BMI), systolic blood pressure (SBP), total cholesterol-to-HDL ratio (CHR), and deprivation)

New: C-index and 95%CI for new model after adding either individual or all physical measures of behaviour, function, and fitness.

Difference: C-index and 95%CI for differences of C-index between base and new model.

Note: 95% confidence intervals for the difference in C-indices were calculated using non-parametric bootstrapping with 1,000 resamples.

**Table S4:** Comparisons of C-index between base models and new models after replacing systolic blood pressure with physical measures of behaviour, function, and fitness

|  |  | **Women** | | **Men** | |
| --- | --- | --- | --- | --- | --- |
| **Physical measure** | **Model** | **Healthy** | **Unhealthy** | **Healthy** | **Unhealthy** |
| Maximum  handgrip  strength | Base | 0.7073 (0.7020, 0.7125) | 0.6963 (0.6884, 0.7042) | 0.7324 (0.7279, 0.7369) | 0.6792 (0.6736, 0.6849) |
|  | New | 0.7079 (0.7026, 0.7131) | 0.7005 (0.6926, 0.7083) | 0.7330 (0.7285, 0.7375) | 0.6844 (0.6788, 0.6900) |
|  | **Difference** | 0.0006 (-0.0001, 0.0014) | 0.0042 (0.0022, 0.0061) | 0.0006 (-0.0002, 0.0015) | 0.0052 (0.0034, 0.0069) |
| Resting  heart  rate | Base | 0.7073 (0.7020, 0.7125) | 0.6963 (0.6884, 0.7042) | 0.7324 (0.7279, 0.7369) | 0.6792 (0.6736, 0.6849) |
|  | New | 0.7094 (0.7042, 0.7147) | 0.7022 (0.6944, 0.7101) | 0.7354 (0.7309, 0.7399) | 0.6842 (0.6785, 0.6899) |
|  | **Difference** | 0.0022 (0.0013, 0.0031) | 0.0059 (0.0037, 0.0082) | 0.0030 (0.0019, 0.0041) | 0.0050 (0.0030, 0.0070) |
| Sleep | Base | 0.7073 (0.7020, 0.7125) | 0.6963 (0.6884, 0.7042) | 0.7324 (0.7279, 0.7369) | 0.6792 (0.6736, 0.6849) |
|  | New | 0.7073 (0.7021, 0.7125) | 0.6992 (0.6914, 0.7070) | 0.7324 (0.7279, 0.7369) | 0.6811 (0.6755, 0.6867) |
|  | **Difference** | 0.0000 (-0.0005, 0.0006) | 0.0029 (0.0012, 0.0046) | 0.0000 (-0.0007, 0.0008) | 0.0019 (0.0005, 0.0033) |
| Leisure  time  physical  activity | Base | 0.7073 (0.7020, 0.7125) | 0.6963 (0.6884, 0.7042) | 0.7324 (0.7279, 0.7369) | 0.6792 (0.6736, 0.6849) |
|  | New | 0.7085 (0.7032, 0.7137) | 0.7036 (0.6958, 0.7115) | 0.7330 (0.7285, 0.7375) | 0.6858 (0.6802, 0.6914) |
|  | **Difference** | 0.0012 (0.0004, 0.0020) | 0.0073 (0.0049, 0.0098) | 0.0006 (-0.0002, 0.0015) | 0.0066 (0.0047, 0.0085) |
| Walking  pace | Base | 0.7073 (0.7020, 0.7125) | 0.6963 (0.6884, 0.7042) | 0.7324 (0.7279, 0.7369) | 0.6792 (0.6736, 0.6849) |
|  | New | 0.7096 (0.7043, 0.7148) | 0.7122 (0.7045, 0.7200) | 0.7339 (0.7294, 0.7385) | 0.6941 (0.6885, 0.6998) |
|  | **Difference** | 0.0023 (0.0013, 0.0033) | 0.0159 (0.0125, 0.0194) | 0.0016 (0.0006, 0.0025) | 0.0149 (0.0123, 0.0176) |
| All physical measures | Base | 0.7073 (0.7020, 0.7125) | 0.6963 (0.6884, 0.7042) | 0.7324 (0.7279, 0.7369) | 0.6792 (0.6736, 0.6849) |
|  | New | 0.7133 (0.7081, 0.7185) | 0.7188 (0.7111, 0.7265) | 0.7403 (0.7358, 0.7447) | 0.7031 (0.6975, 0.7088) |
|  | **Difference** | 0.0061 (0.0046, 0.0075) | 0.0225 (0.0184, 0.0266) | 0.0079 (0.0063, 0.0094) | 0.0239 (0.0208, 0.0270) |

Base: C-index and 95%CI for base model (age, smoking status, body mass index (BMI), systolic blood pressure (SBP), total cholesterol-to-HDL ratio (CHR), and deprivation)

New: C-index and 95%CI for new model after adding either individual or all physical measures of behaviour, function, and fitness.

Difference: C-index and 95%CI for differences of C-index between base and new model.

Note: 95% confidence intervals for the difference in C-indices were calculated using non-parametric bootstrapping with 1,000 resamples.

**Table S5:** Comparisons of C-index between base models and new models after replacing both cholesterol-to-HDL ratio and systolic blood pressure with physical measures of behaviour, function, and fitness

|  |  | **Women** | | **Men** | |
| --- | --- | --- | --- | --- | --- |
| **Physical measure** | **Model** | **Healthy** | **Unhealthy** | **Healthy** | **Unhealthy** |
| Maximum  handgrip  strength | Base | 0.7073 (0.7020, 0.7125) | 0.6963 (0.6884, 0.7042) | 0.7324 (0.7279, 0.7369) | 0.6792 (0.6736, 0.6849) |
|  | New | 0.7075 (0.7022, 0.7127) | 0.6998 (0.6919, 0.7076) | 0.7320 (0.7276, 0.7365) | 0.6837 (0.6781, 0.6893) |
|  | **Difference** | 0.0002 (-0.0006, 0.0010) | 0.0035 (0.0014, 0.0056) | -0.0003 (-0.0013, 0.0007) | 0.0045 (0.0026, 0.0064) |
| Resting  heart  rate | Base | 0.7073 (0.7020, 0.7125) | 0.6963 (0.6884, 0.7042) | 0.7324 (0.7279, 0.7369) | 0.6792 (0.6736, 0.6849) |
|  | New | 0.7092 (0.7039, 0.7144) | 0.7015 (0.6936, 0.7094) | 0.7347 (0.7302, 0.7391) | 0.6836 (0.6779, 0.6893) |
|  | **Difference** | 0.0019 (0.0009, 0.0029) | 0.0052 (0.0029, 0.0075) | 0.0023 (0.0011, 0.0035) | 0.0044 (0.0023, 0.0064) |
| Sleep | Base | 0.7073 (0.7020, 0.7125) | 0.6963 (0.6884, 0.7042) | 0.7324 (0.7279, 0.7369) | 0.6792 (0.6736, 0.6849) |
|  | New | 0.7069 (0.7017, 0.7121) | 0.6985 (0.6907, 0.7063) | 0.7315 (0.7270, 0.7360) | 0.6804 (0.6747, 0.6860) |
|  | **Difference** | -0.0004 (-0.0011, 0.0003) | 0.0022 (0.0004, 0.0040) | -0.0009 (-0.0018, -0.0000) | 0.0011 (-0.0004, 0.0026) |
| Leisure  time  physical  activity | Base | 0.7073 (0.7020, 0.7125) | 0.6963 (0.6884, 0.7042) | 0.7324 (0.7279, 0.7369) | 0.6792 (0.6736, 0.6849) |
|  | New | 0.7081 (0.7029, 0.7133) | 0.7029 (0.6950, 0.7108) | 0.7321 (0.7276, 0.7366) | 0.6850 (0.6794, 0.6906) |
|  | **Difference** | 0.0008 (-0.0001, 0.0017) | 0.0066 (0.0040, 0.0092) | -0.0003 (-0.0013, 0.0007) | 0.0058 (0.0037, 0.0078) |
| Walking  pace | Base | 0.7073 (0.7020, 0.7125) | 0.6963 (0.6884, 0.7042) | 0.7324 (0.7279, 0.7369) | 0.6792 (0.6736, 0.6849) |
|  | New | 0.7092 (0.7040, 0.7144) | 0.7117 (0.7039, 0.7195) | 0.7331 (0.7286, 0.7377) | 0.6935 (0.6879, 0.6992) |
|  | **Difference** | 0.0019 (0.0009, 0.0030) | 0.0154 (0.0119, 0.0190) | 0.0008 (-0.0003, 0.0019) | 0.0143 (0.0116, 0.0170) |
| All physical measures | Base | 0.7073 (0.7020, 0.7125) | 0.6963 (0.6884, 0.7042) | 0.7324 (0.7279, 0.7369) | 0.6792 (0.6736, 0.6849) |
|  | New | 0.7131 (0.7079, 0.7183) | 0.7183 (0.7106, 0.7260) | 0.7397 (0.7352, 0.7442) | 0.7026 (0.6970, 0.7083) |
|  | **Difference** | 0.0059 (0.0044, 0.0073) | 0.0220 (0.0178, 0.0262) | 0.0074 (0.0057, 0.0090) | 0.0234 (0.0203, 0.0266) |

Base: C-index and 95%CI for base model (age, smoking status, body mass index (BMI), systolic blood pressure (SBP), total cholesterol-to-HDL ratio (CHR), and deprivation)

New: C-index and 95%CI for new model after adding either individual or all physical measures of behaviour, function, and fitness.

Difference: C-index and 95%CI for differences of C-index between base and new model.

Note: 95% confidence intervals for the difference in C-indices were calculated using non-parametric bootstrapping with 1,000 resamples.

**Table S6:** Net Reclassification Indices for 10-year survival probability prediction comparing the base model with the new model with physical behaviour, physical function, and fitness measures in each step.

|  |  | **Women** | | **Men** | |
| --- | --- | --- | --- | --- | --- |
| **Step** | **Physical measure** | **Healthy** | **Unhealthy** | **Healthy** | **Unhealthy** |
| **Addition** | Maximum handgrip strength | -0.0056 (-0.0130, 0.0018) | 0.0229 (0.0043, 0.0414) | -0.0054 (-0.0139, 0.0031) | 0.0512 (0.0349, 0.0675) |
|  | Resting heart rate | -0.0134 (-0.0213, -0.0054) | 0.0193 (-0.0015, 0.0401) | -0.0075 (-0.0156, 0.0007) | 0.0488 (0.0324, 0.0651) |
|  | Sleep | -0.0037 (-0.0095, 0.0020) | 0.0049 (-0.0096, 0.0195) | -0.0010 (-0.0066, 0.0046) | 0.0172 (0.0068, 0.0277) |
|  | Leisure time physical activity | -0.0104 (-0.0184, -0.0025) | 0.0389 (0.0139, 0.0639) | -0.0040 (-0.0121, 0.0040) | 0.0769 (0.0566, 0.0973) |
|  | Walking pace | -0.0165 (-0.0244, -0.0087) | 0.1202 (0.0842, 0.1563) | 0.0169 (0.0051, 0.0287) | 0.1582 (0.1339, 0.1825) |
|  | All physical measures | -0.0298 (-0.0405, -0.0191) | 0.1020 (0.0714, 0.1326) | 0.0017 (-0.0099, 0.0133) | 0.2020 (0.1793, 0.2247) |
| **Substitution of Cholesterol-to-HDL Ratio** | Maximum handgrip strength | -0.0065 (-0.0143, 0.0012) | 0.0233 (0.0040, 0.0426) | -0.0034 (-0.0125, 0.0056) | 0.0436 (0.0267, 0.0606) |
|  | Resting heart rate | -0.0139 (-0.0222, -0.0055) | 0.0248 (0.0038, 0.0458) | -0.0066 (-0.0154, 0.0023) | 0.0469 (0.0304, 0.0634) |
|  | Sleep | -0.0035 (-0.0101, 0.0032) | 0.0132 (-0.0013, 0.0278) | -0.0028 (-0.0102, 0.0046) | 0.0161 (0.0041, 0.0280) |
|  | Leisure time physical activity | -0.0128 (-0.0208, -0.0048) | 0.0387 (0.0138, 0.0636) | -0.0065 (-0.0147, 0.0017) | 0.0754 (0.0545, 0.0964) |
|  | Walking pace | -0.0120 (-0.0201, -0.0039) | 0.1115 (0.0752, 0.1479) | 0.0152 (0.0033, 0.0271) | 0.1543 (0.1295, 0.1792) |
|  | All physical measures | -0.0276 (-0.0381, -0.0171) | 0.0973 (0.0665, 0.1281) | -0.0006 (-0.0129, 0.0118) | 0.1963 (0.1734, 0.2193) |
| **Substitution of Systolic Blood Pressure** | Maximum handgrip strength | -0.0015 (-0.0101, 0.0070) | 0.0134 (-0.0053, 0.0321) | 0.0016 (-0.0072, 0.0103) | 0.0405 (0.0231, 0.0579) |
|  | Resting heart rate | -0.0079 (-0.0171, 0.0013) | 0.0217 (0.0010, 0.0423) | -0.0012 (-0.0102, 0.0078) | 0.0373 (0.0205, 0.0542) |
|  | Sleep | 0.0013 (-0.0059, 0.0085) | 0.0076 (-0.0075, 0.0227) | 0.0044 (-0.0040, 0.0128) | 0.0067 (-0.0055, 0.0189) |
|  | Leisure time physical activity | -0.0067 (-0.0158, 0.0024) | 0.0379 (0.0131, 0.0628) | 0.0023 (-0.0065, 0.0112) | 0.0659 (0.0454, 0.0864) |
|  | Walking pace | -0.0132 (-0.0220, -0.0045) | 0.1122 (0.0763, 0.1481) | 0.0229 (0.0106, 0.0352) | 0.1481 (0.1235, 0.1727) |
|  | All physical measures | -0.0255 (-0.0367, -0.0142) | 0.0989 (0.0684, 0.1295) | 0.0068 (-0.0052, 0.0188) | 0.1939 (0.1708, 0.2171) |
| **Substitution of both Cholesterol-to-HDL Ratio and systolic blood pressure** | Maximum handgrip strength | -0.0019 (-0.0111, 0.0074) | 0.0150 (-0.0043, 0.0343) | 0.0009 (-0.0084, 0.0101) | 0.0366 (0.0189, 0.0543) |
|  | Resting heart rate | -0.0094 (-0.0187, -0.0000) | 0.0232 (0.0025, 0.0438) | 0.0018 (-0.0075, 0.0110) | 0.0337 (0.0163, 0.0512) |
|  | Sleep | 0.0039 (-0.0038, 0.0116) | 0.0103 (-0.0053, 0.0260) | 0.0051 (-0.0039, 0.0141) | 0.0032 (-0.0106, 0.0170) |
|  | Leisure time physical activity | -0.0079 (-0.0171, 0.0013) | 0.0353 (0.0104, 0.0602) | 0.0032 (-0.0058, 0.0123) | 0.0629 (0.0422, 0.0837) |
|  | Walking pace | -0.0112 (-0.0204, -0.0021) | 0.1097 (0.0737, 0.1456) | 0.0209 (0.0084, 0.0334) | 0.1397 (0.1147, 0.1648) |
|  | All physical measures | -0.0246 (-0.0360, -0.0133) | 0.0964 (0.0653, 0.1275) | 0.0051 (-0.0070, 0.0171) | 0.1901 (0.1664, 0.2137) |

**Figure S1**: Kaplan–Meier survival curves comparing survival probability across stratification on sex and health conditions.

*
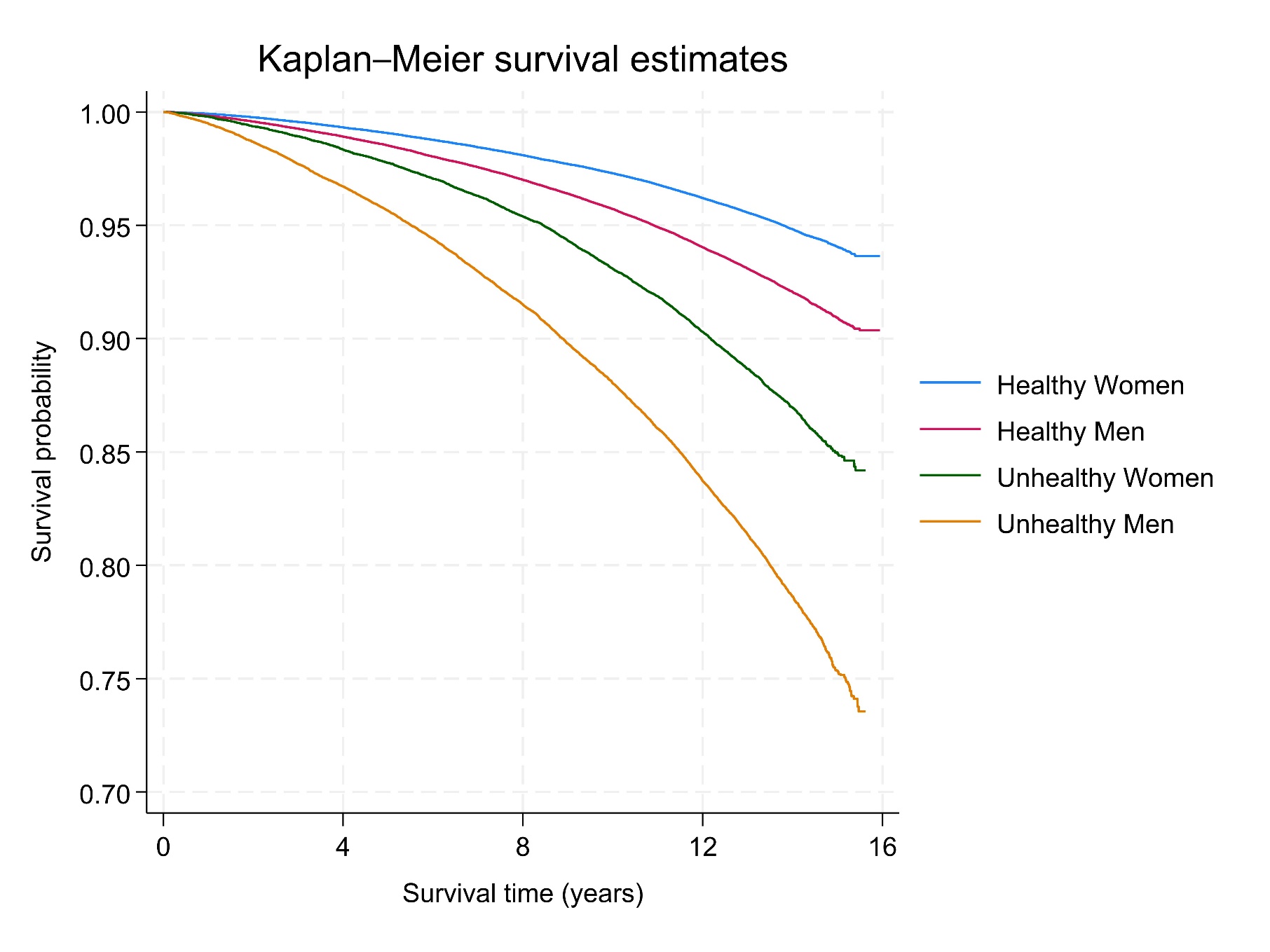
*

**Figure S2**: Sensitivity analyses on C-index in people with CVD and cancer comparing base models versus models with added or substituted physical behaviour, function, and fitness measures


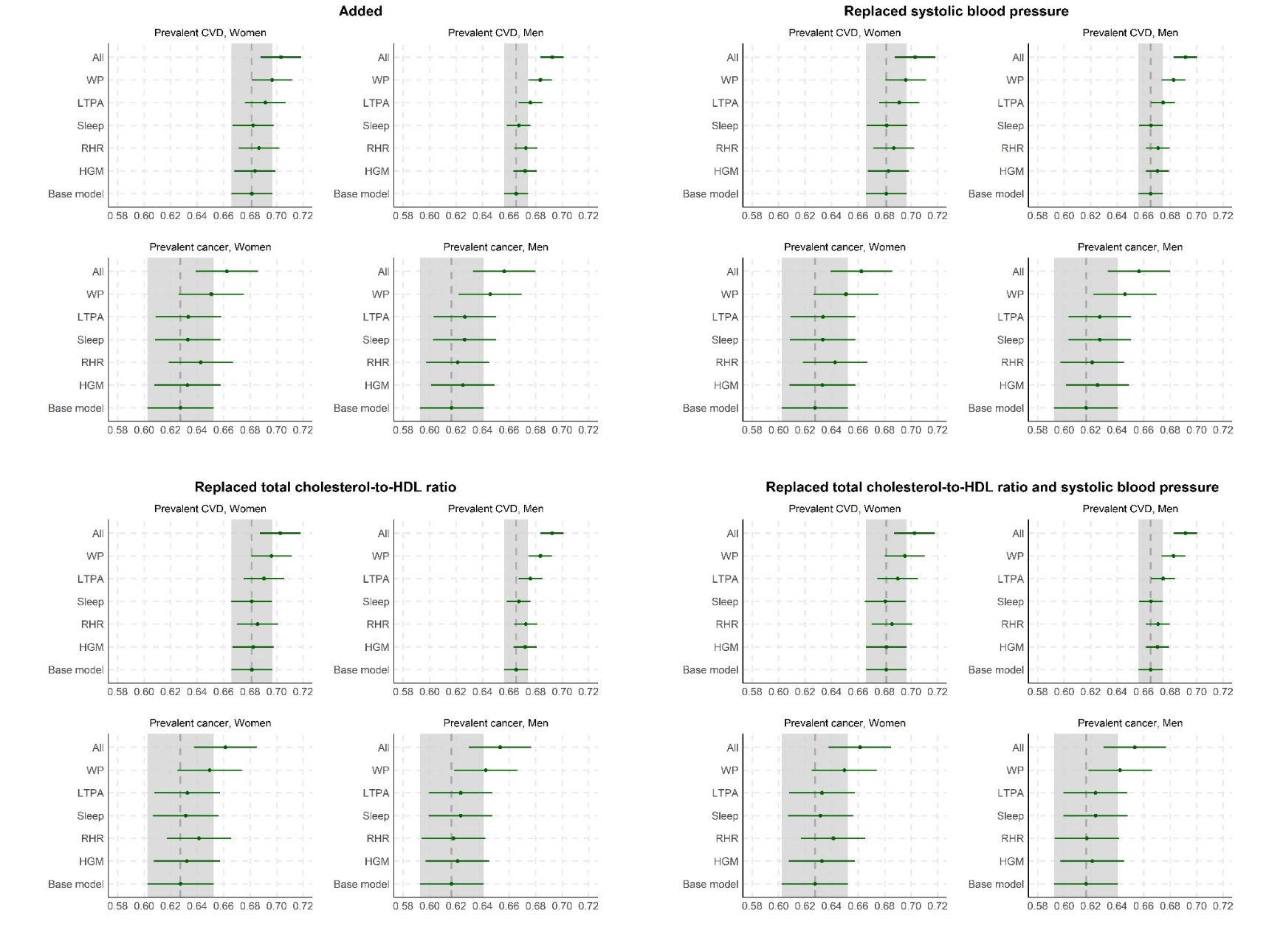


*Base models included: age, smoking status, BMI, SBP, CHR, and deprivation*

*WP: walking pace*

*LTPA: leisure time physical activity*

*RHR: resting heart rate*

*HGM: handgrip strength (maximum)*

*All: base model covariates and 5 physical behaviour, fitness and function indicators (WP, LTPA, RHR, HGM and sleep)*

*BMI: Body Mass Index*

*SBP: Systolic Blood Pressure*

*CHR: Total Cholesterol to high-density lipoprotein Cholesterol Ratio*

**Figure S3:** Sensitivity analyses of the C-index in participants excluding those who died within the first two years, comparing base models with models that included or substituted physical behaviour, function, and fitness measures.


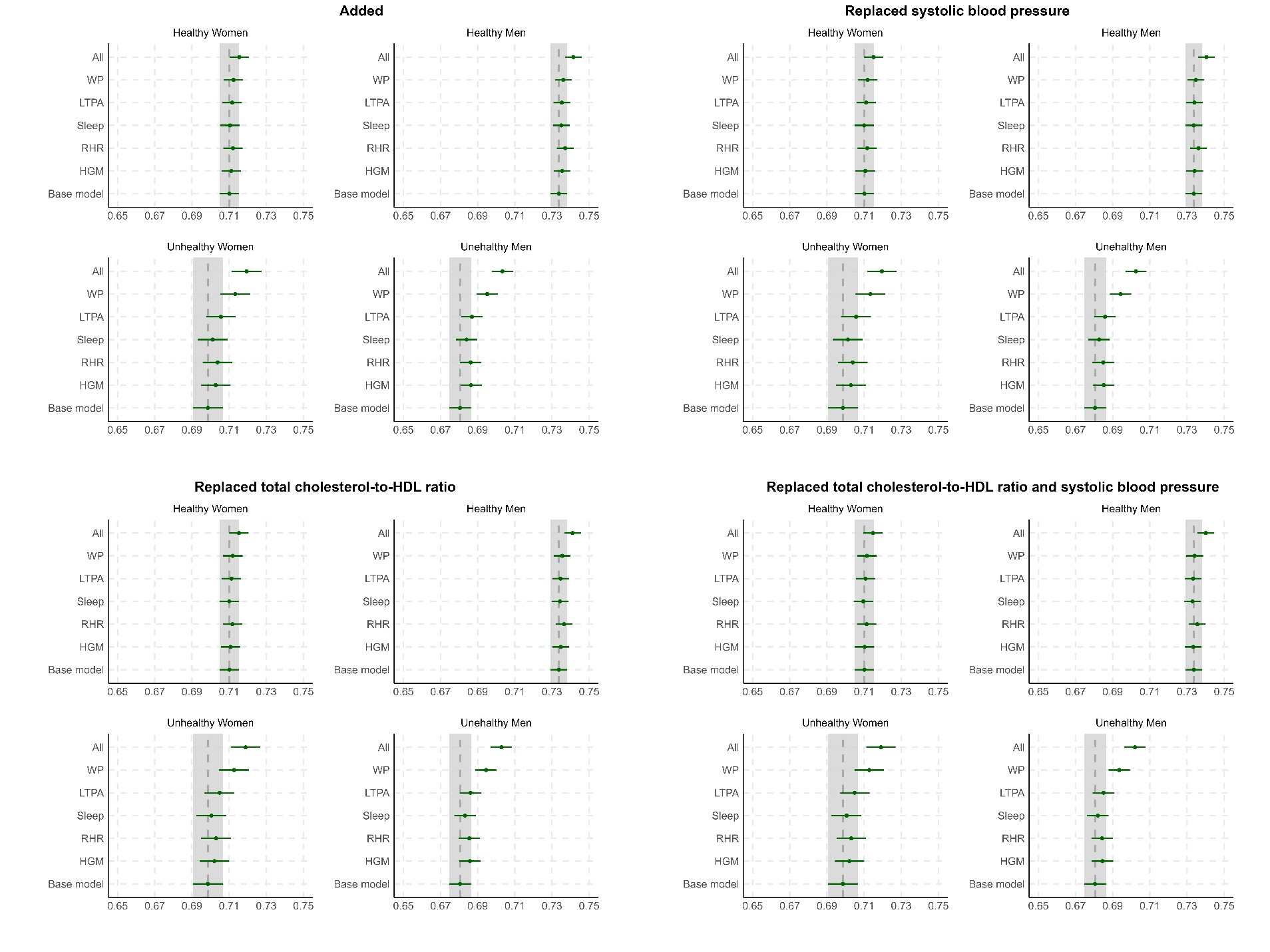


*Base models included: age, smoking status, BMI, SBP, CHR, and deprivation*

*WP: walking pace*

*LTPA: leisure time physical activity*

*RHR: resting heart rate*

*HGM: handgrip strength (maximum)*

*All: base model covariates and 5 physical behaviour, fitness and function indicators (WP, LTPA, RHR, HGM and sleep)*

*BMI: Body Mass Index*

*SBP: Systolic Blood Pressure*

*CHR: Total Cholesterol to high-density lipoprotein Cholesterol Ratio*
